# Aromatase inhibition rapidly affects in a reversible manner distinct features of birdsong

**DOI:** 10.1038/srep32344

**Published:** 2016-08-30

**Authors:** Beau A. Alward, Catherine de Bournonville, Trevor T. Chan, Jacques Balthazart, Charlotte A. Cornil, Gregory F. Ball

**Affiliations:** 1 Department of Psychological and Brain Sciences, Johns Hopkins University, 3400 North Charles Street, Baltimore, MD, 21218, USA; 2 GIGA Neuroscience, University of Liege, Avenue Hippocrate, 15, 4000 Liege, Belgium, USA

## Abstract

Recent evidence has implicated steroid hormones, specifically estrogens, in the rapid modulation of cognitive processes. Songbirds have been a useful model system in the study of complex cognitive processes including birdsong, a naturally learned vocal behavior regulated by a discrete steroid-sensitive telencephalic circuitry. Singing behavior is known to be regulated by long-term actions of estrogens but rapid steroid modulation of this behavior has never been examined. We investigated if acute actions of estrogens regulate birdsong in canaries (*Serinus canaria*). In the morning, male canaries sing within minutes after light onset. Birds were injected with fadrozole, a potent aromatase inhibitor, or vehicle within 2–5 minutes after lights on to implement a within-subjects experimental design. This single injection of fadrozole reduced the motivation to sing as well as song acoustic stereotypy, a measure of consistency over song renditions, on the same day. By the next day, however, all song measures that were affected had returned to baseline. This study indicates that estrogens also act in a rapid fashion to regulate two distinct features of song, a learned vocal behavior.

Behavioral and cognitive processes have been shown to be modulated by steroid hormones^1^. This modulation of behavior by steroid hormones can occur through both genomic and non-genomic actions, although evidence for the latter has begun to accumulate only relatively recently^2^. For instance, in male Japanese quail (*Coturnix japonica*) and female Long Evans rats (*Rattus norvegicus*) estrogens have been shown to facilitate sociosexual behaviors in a rapid, short-lasting (i.e., acute) manner^3^,^4^,^5^. Aggressive behavior in song sparrows (*Melospiza melodia*) has also been shown to be rapidly regulated by estrogens: a single injection of the aromatase inhibitor fadrozole decreased within a day territorial aggression during the non-breeding season^6^. There is also recent evidence that cognitive processes like memory are similarly under the control of the acute actions of steroid hormones^7^,^8^ but our understanding of the role of these acute actions of steroid hormones remains incomplete. Birdsong is an excellent model system for the study of complex cognitive processes^9^. Songbirds naturally learn their song in a manner akin to how humans learn speech^10^,^11^. The production of song relies on the interaction between multiple functionally-discrete telencephalic nuclei (collectively called the song control system or SCS) as well as areas involved in motivation, providing an opportunity to study complex cognitive processes with multiple, distinct features. Moreover, multiple sites for the actions of steroid hormones have been identified throughout the SCS^12^. Therefore, it is especially useful to investigate the modulation by steroid hormones of these behaviors in songbirds.

Birdsong is well known to be regulated by steroid hormones such as testosterone and its metabolites^12^,^13^. Castration in canaries (*Serinus canaria*) substantially reduces song output and treatment with exogenous testosterone restores singing after approximately three days^14^,^15^,^16^. In zebra finches (*Taeniopygia guttata*) chronic treatment with ATD (1,4,6-androstatriene-3,17-dione), a potent inhibitor of aromatase, the enzyme that converts testosterone to estradiol (E2), reduces song output^17^. Androgen receptors are expressed in the telencephalic song control nuclei HVC, RA, LMAN, and in a variety of nuclei in the hypothalamus and midbrain and estrogen receptors (ER) are expressed in HVC (ER alpha) in some songbird species as well as in the hypothalamus (both ER alpha and beta)^18^,^19^. Aromatase is also widely distributed throughout the songbird brain particularly in the hypothalamus and the preoptic area (POA) including the medial preoptic nucleus (POM)^20^, as well as in non-hypothalamic areas such as the dorsal telencephalon^20^,^21^,^22^. This distribution is generally consistent with what is observed in rodent species^23^,^24^. However, aromatase activity is in general much higher in the songbird brain^25^ and there is, contrary to what is observed in rodents, an especially high degree of aromatase activity in the telencephalon^26^,^27^,^28^. Aromatase is noticeably absent from cell bodies in song control regions such as HVC and RA, but densely expressed in the auditory regions adjacent to HVC, such as the caudomedial nidopallium (NCM) and caudomedial mesopallium (CMM). Specifically, aromatase is expressed in NCM neurons in both cell bodies and presynaptic terminals, and in presynaptic terminals in HVC^29^,^30^.

This presynaptic distribution of aromatase in HVC suggests that birdsong, a learned vocal behavior, may be under the control of the acute actions of steroid hormones such as estrogens. In the current study, we investigate in a well-studied songbird species, the canary, the role of the fast actions of estrogens in the regulation of birdsong.

## Results

### Effects of aromatase inhibition on song

The descriptive statistics of all song measures and the result of their statistical analyses are presented in Table 1.

### Acute aromatase inhibition leads to a decrease in the motivational measures of song

**Figure 1:**
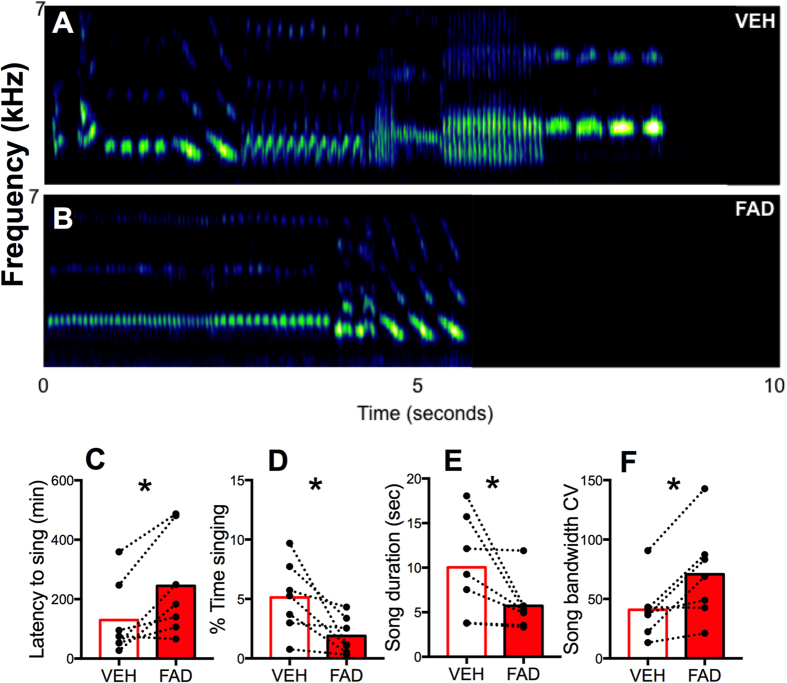
Effects of acute aromatase inhibition with fadrozole on multiple measures of song. Representative songs from a bird treated with (**A**) vehicle (VEH) versus (**B**) fadrozole (FAD). Treatment affected measures of the motivation to sing including (**C**) the Latency to sing, (**D**) % Time singing, (**E**) Song duration, and a measure of song stereotypy (**F**) Song bandwidth coefficient of variation (CV). The higher the CV, the lower the stereotypy and vice versa. Bars represent the mean of all data in the corresponding group. Asterisks indicate a significant difference. Differences were considered significant at p < 0.05.

Three birds did not sing at all on the day they were injected with vehicle and 5 did not sing on the day they were injected with fadrozole (Fisher exact probability test: p = 0.67). Only birds that sang on each injection day were included in these analyses (See ‘Methods: Song recording and analysis’). Representative songs are shown for a bird treated with vehicle versus fadrozole in Fig. 1 (Fig. 1A,B). On the day of injections, birds treated with fadrozole started singing after significantly longer latencies following injection than birds treated with vehicle (Fig. 1C; t_6_ = 3.44, p < 0.05, d = 1.30). Fadrozole-treated birds also spent less time singing (Fig. 1D; t_6_ = 2.98, p < 0.05, d = 1.13) and sang shorter songs (Fig. 1E; t_6_ = 2.75, p < 0.05, d = 1.04).

### Acute aromatase inhibition reduces song stereotypy

Fadrozole treatment also caused a decrease in the stereotypy of song as evident based on the fact that birds treated with fadrozole sang songs with higher bandwidth CV (Fig. 1F; t_6_ = 3.49, p < 0.05, d = 1.31) (higher CV = lower stereotypy).

All significant differences concerning singing motivation and song stereotypy between the fadrozole and vehicle conditions were associated with large effect sizes as reflected by the Cohen’s d values larger than 1 in each case (see Table).

### The effects of acute aromatase inhibition on song disappeared the day after treatment

**Figure 2:**
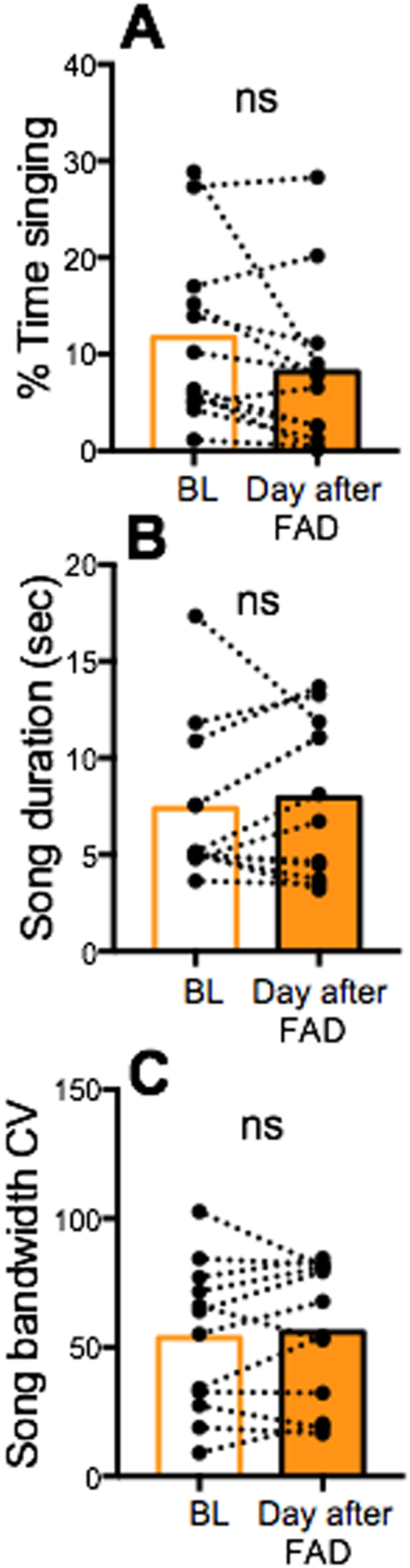
Comparison of song features reflecting the motivation to sing (**A,B**) and the song stereotypy (**C**) on the baseline (BL) day and on the day after fadrozole (FAD) injection. CV = Coefficient of Variation. The higher the CV, the lower the stereotypy and vice versa. Bars represent the mean of all data in the corresponding group. In all cases differences were non-significant (ns).

All features of song that were affected on the day of fadrozole treatment were back to normal levels the day after treatment (Fig. 2; t_11_ = 0.71, p ≥ 0.49 for all comparisons except for % time spent singing where a statistical tendency was still present; t_11_ = 2.05, p = 0.07).

### Effects of fadrozole on aromatase activity

**Figure 3:**
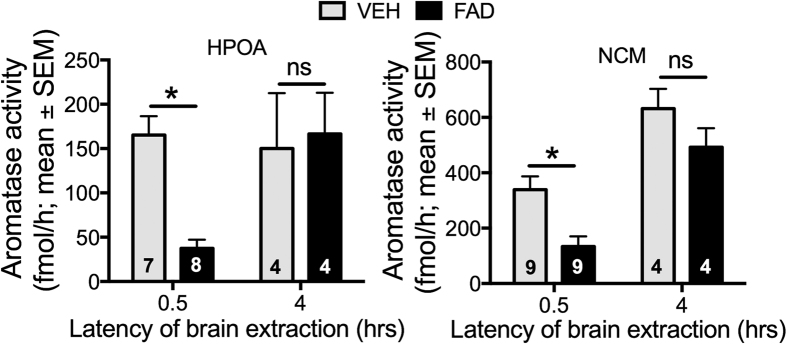
Effects of fadrozole on aromatase activity (AA) at two different time points after injection. Fmol/h = fentomoles per hour. The numbers within each bar represent sample size. Asterisks indicate a significant difference between the AA in the respective brain regions as indicated by Bonferroni-corrected planned comparisons at the different time points. Bars represent the means ± standard errors. Differences were considered significant at p < 0.05.

Average levels of aromatase activity at 30 min or 4 hours after a fadrozole or control injection are shown in Fig. 3. Values for aromatase activity in the 30-min HPOA group of one vehicle-treated and one fadrozole-treated birds were outliers (see ‘Methods’) and these birds were removed from the analysis of aromatase activity in the 30-min HPOA group (see Fig. 3 for final sample sizes per group). In the HPOA, after thirty minutes, fadrozole treatment led to a substantial reduction of aromatase activity compared to vehicle (Bonferroni’s, t_13_ = 3.40, p < 0.05); however, this difference had disappeared four hours after injection (Bonferroni’s, t_6_ = 0.35, p = 0.75). In the NCM, 30 minutes after injection fadrozole similarly caused a large reduction in aromatase activity relative to vehicle (Bonferroni’s, t_16_ = 3.32, p < 0.01), whereas this difference was no longer present at four hours after injection (Bonferroni’s, t_6_ = 1.50, p = 0.18).

## Discussion

Many studies on the hormonal regulation of birdsong have focused on investigations of the long-term effects of sex steroid hormones (e.g., see^13^ for a review). Recently, evidence has begun to accumulate indicating that certain behaviors and cognitive processes may be regulated by estrogens acting in a much faster, presumably non-genomic, fashion^3^,^4^,^5^,^7^,^31^. Moreover, song sparrows show enhanced concentrations of estrogens in the brain in the breeding season versus the non-breeding season^32^ and estradiol increases aggression within 20 minutes in this species^33^. The experiment presented here suggests that estrogens may act in this fashion in the regulation of a complex, learned vocal behavior.

Our experiment demonstrates that acute aromatase inhibition causes a variety of song features to undergo rapid and prominent changes. A single fadrozole injection decreased motivational measures of song and decreased song stereotypy on the day of treatment and all of the affected measures returned to baseline by the next day. These results suggest that aromatase present in different brain regions plays an important role in regulating these distinct features of song. For instance, as mentioned above, aromatase is densely expressed in the POM of zebra finches and the conversion of testosterone to E2 is required for the full activation of singing behavior in this species^17^,^20^,^34^. These observations suggest that one possible site at which the acute inhibition of aromatase causes the observed reduction in the motivation to sing is the POM. Indeed, work in male Japanese quail has shown that estrogens act in an acute manner in the brain to activate motivational aspects of male-typical sexual behaviors^3^,^4^,^35^ and based on the extensive work on male sexual behavior in this species, a likely site for these rapid actions of estrogens in the regulation of motivation is the POM^36^,^37^,^38^. Work in canaries and starlings supports the contention that aromatase in POM is critical for the rapid changes in the motivation to sing observed in the current study. For instance, lesions to POM cause substantial reductions in the motivation to sing^39^ and testosterone implanted solely in POM of castrated canaries enhances the motivation to sing in the absence of increases in song stereotypy^14^,^15^. Testosterone implanted in the POM of castrated canaries took approximately 7 days to fully enhance the motivation to sing^15^, suggesting the effects were genomic in nature. It has been established that testosterone enhances the expression and activity of aromatase in the preoptic area of songbirds^40^,^41^ and the increased expression of the corresponding gene in the POM might represent one of the genomic effects underlying song activation. As a consequence, the POM would then be a plausible site where locally produced neuroestrogens act to regulate the motivation to sing^5^. Part of these effects could additionally be rapid and mediated by non-genomic regulations of aromatase activity^42^,^43^,^44^,^45^.

However, the relatively rapid changes in song stereotypy are unlikely to be controlled by changes in aromatase activity or the activation of ER in the POM. ER Activation in the SCS has been shown to enhance song stereotypy. For instance, blocking ER in the HVC of white crowned sparrows via the chronic infusion of the ER antagonist tamoxifen reduced song acoustic stereotypy^46^. In our experiment, blocking aromatase activity likely led to an acute decrease in E2 concentrations acting in HVC, thus causing a reduction in song acoustic stereotypy. The source of E2 could have been the testis or E2 generated in the brain itself (i.e., neuroestrogens). Indeed, while aromatase is not expressed in HVC itself, it is expressed at high levels in the nidopallium surrounding HVC. For instance, neurons in NCM, an auditory region within the nidopallium, express aromatase^20^ and those aromatase-expressing neurons project to HVC^21^. Remage-Healey and colleagues^47^ have shown that E2 produced in NCM enhances the selectivity of HVC neurons to the bird’s own song (BOS). The depletion of E2 production caused by fadrozole injection may have caused disruptions in BOS selectivity, which could have led to decreased song stereotypy. Hence, there are multiple ways by which the inhibition of aromatase could have led to decreases in song stereotypy.

Finally, recent work suggests there may be a link between the actions of steroid hormones and regulation of speech production and vocal plasticity in humans^48^,^49^. Work in songbirds has been critical in providing possible causal mechanisms for steroid hormones in the regulation of vocal plasticity^14^,^15^,^46^,^50^,^51^,^52^. However, all of these studies used time scales that were far too long (i.e., days to weeks) to elucidate the acute actions of steroid hormones in regulating vocal plasticity. The results presented here provide evidence that the motivation to produce learned vocalizations and vocal plasticity itself are mediated by acute actions of steroid hormones. Song control in canaries might thus be under steroid control both in the long- and in the short-term which is consistent with the recently proposed dual action hypothesis of estrogen action^2^. This raises the intriguing question of whether changes in vocal plasticity in humans may also be regulated by acute actions of steroid hormones.

## Methods

### Animals and pre-experimental manipulations

We used 12 canaries of the American singer strain because preliminary studies from our laboratory had found that they sing readily after handling and other non-invasive manipulations. Birds were obtained from a local breeder (Maryland Exotic Birds). Upon entry into the lab birds were placed on a short day (SD) photoperiod (8 L:16D) for six weeks to maintain photosensitivity^53^. The protocols and procedures used here were approved by the Johns Hopkins University Animal Care and Use Committee (protocol number: AV14A112) and followed the ASAB/ABS Guidelines for the use of animals in research.

### Acclimation and injection procedures

Birds were placed in sound-attenuated, isolation chambers (41 cm × 48 cm × 51 cm) set to long days (14L:10D) to simulate breeding conditions^53^,^54^. We randomly selected three groups of four birds to experience lights on at either 08:00 h, 08:02 h, or 08:04 h which experienced lights off at 22:00 h, 22:02 h, or 22:04 h, respectively. This was done to ensure all birds were injected during the same relative time frame following lights on. Seven days later birds were handled and injected with vehicle propylene glycol (propylene glycol:saline = 4:1) 2–5 minutes after lights on to simulate the handling associated with the injections and habituate birds to the injection procedure. Injections were made intraperitoneally by using forceps to lift up the skin above the abdomen and inserting the needle and immediately making the injection. One person would grab the bird from its cage 2–5 minutes after lights on and hold it in their hand while another person made the injection. The whole injection procedure took less than a minute in almost all cases.

### Procedures

Three days after the acclimation period, between two and five minutes after lights on, birds were either injected with fadrozole (Fadrozole hydrochloride, Sigma Aldrich F3806; 30 mg/kg; (n = 6) dissolved in propylene glycol:saline (4:1) or with vehicle only (n = 6). The dose of fadrozole was selected based on previous studies in fish, birds and mammals demonstrating rapid behavioral effects of this dose of fadrozole or of the related and similar inhibitor vorozole^35^,^55^,^56^ (See ref. 52 for a comparison of effective doses producing acute effects in various animal models). This dose of vorozole had also been shown to completely inhibit with 30 min brain aromatase activity in quail^35^. Three days later these injections were repeated but the subjects assigned to each treatment were reversed so that each bird had been subjected to both the vehicle and fadrozole treatment. Four days following the second injection, birds were injected again with either vehicle or fadrozole and their brains were extracted 30 minutes or 4 hours later (see ‘Brain and Blood Collection’ below).

Male canaries sing prolifically within minutes following light on^14^,^15^,^54^,^57^,^58^,^59^. However, based on pilot studies, injections (using vehicle) can delay the onset of singing behavior for multiple hours. Therefore, to increase the likelihood that samples of song from each bird could be captured on the day of injection, song was recorded on these days from lights on (800 h) to 1200 h and from 1300 h to 1600 h. The day before the first injection, song was recorded from 800 h to 1030 h to provide a baseline level of singing^14^,^15^,^54^,^57^. On the day following the injection of fadrozole, song was also recorded for 800 h to 1030 h. These various recording procedures were designed to answer two questions: 1) How is song affected on the fadrozole injection day as compared to song recorded on the day when only the vehicle was injected? and 2) Are any of the observed changes still present on the following day?

### Song recording and analysis

On the first day of the acclimation period, birds were placed individually in sound-attenuating recording chambers (41 cm × 48 cm × 51 cm). Isolation chambers were outfitted with a microphone (BT-MP8087 Mini microphone; B&H Photo and Electronics Corp, New York, NY) and camera (KPC-600 Pinhole Camera 3.6 mm; B&H Photo and Electronics Corp, New York, NY) connected to a computer running DVRserver (V6.33b; Mammoth Technologies, Austin, TX) designed for real-time video and audio surveillance recording. The DVRserver captured song behavior. Recordings were converted to.wav files sampled at 22,050 Hz which translated to a frequency range of 0–11 kHz. Song files were run through a high-pass filter set to a threshold of 900 Hz to remove low-frequency noise and converted to a digital format using Goldwave™ (Version 5.55; GoldWave, St. John’s, NF, Canada) before they were visualized into sound spectrograms using Avisoft (SASlab Pro, Berlin, Germany), a Windows application for investigating animal acoustic communication. For the spectrograms, the fast Fourier transform length was set to 512 with an overlap of 75% for the temporal resolution. Songs were defined as vocalizations that have a duration >1 second of continuous vocalizations with gaps no longer than 500 milliseconds^14^,^15^,^54^,^57^,^60^,^61^. Each song was verified by looking at the original sonograms to further eliminate noise and false positives that escaped the filter.

Based on previous work, we used Avisoft to quantify the following song features: latency to sing following injection, % time singing, mean song duration–three measures of the motivation to sing–and song acoustic stereotypy^14^,^15^,^46^,^54^,^59^. We calculated % time singing by dividing the total time each bird spent singing by the total sampling time on each day and multiplying this value by 100. Mean song duration was calculated by averaging across each song on each day. We predicted that inhibiting aromatase with fadrozole would cause reductions in both the motivation to sing^3^,^4^,^17^,^35^ as well as song stereotypy^46^. We used Avisoft to quantify song stereotypy. Our previous work as well as the work of others has shown that in canaries the actions of testosterone and its metabolites are critical for enhancing song acoustic stereotypy^14^,^15^,^46^. We used Avisoft to quantify the bandwidth for each song, and from this we computed song bandwidth stereotypy. Song bandwidth stereotypy was chosen based on previous work^14^,^15^. For each recording day (baseline day, injection day, day after injection), average (AVG) bandwidth and the associated standard deviation (SD) were computed over all songs on those specific days. The stereotypy of bandwidth was computed using the coefficient of variation (CV) (CV = (SD/AVG)*100)); the higher the CV of song bandwidth, the lower the stereotypy of said. CV of acoustic variables has been used in previous studies as measures of song stereotypy^14^,^15^,^46^,^59^,^62^,^63^.

It should be noted that some birds did not sing at all on a given day. Because the experiment used a within-subjects design (birds compared to themselves in different conditions) and the reasons potentially explaining this inactivity can be variable including but not limited to the aromatase inhibition after fadrozole injection, these birds where no recording was available for a given day (after injection of either fadrozole or vehicle) had to be removed from the analyses (See beginning of the ‘Results’ section for exact numbers). This explains why the degrees of freedom in the results are often smaller than the number of birds actually included in the experiment.

### Brain and blood collection

Four days after the last injection of this experiment, birds (12 males from this experiment and 16 additional males) were injected within 2–5 minutes after lights on with vehicle (n = 14) or fadrozole (n = 14). Their brains were rapidly extracted 30 minutes (10 from each group) or 4 hours (4 from each group) later and frozen on dry ice. One bird from the 30-minute group that was injected with vehicle and one bird of the 30-minute group injected with fadrozole escaped and flew around for an extended period of time before or after being injected and they were consequently excluded from the analysis of aromatase activity reducing the number of available brains to 9 in these two groups. We ensured not to give birds a fadrozole injection on the day of brain extraction if they had received an experimental injection of fadrozole four days earlier to minimize possible carry-over effects. Brains remained on dry ice for at least five minutes before being stored at −70 °C until assessment of aromatase activity (see above).

These brains were used to assess the efficacy of fadrozole on inhibiting brain aromatase (see below). The 30-minute time point was chosen based on observations in a previous study in male Japanese quail that showed Vorozole**™** (another very similar aromatase inhibitor) caused a substantial reduction in aromatase activity 30 minutes after it was injected^35^. The 4-hour time point was chosen based on behavioral observations from this experiment that most birds treated with fadrozole begin showing a rebound in song behavior after about 4 hours, suggesting aromatase activity has returned to normal levels.

### Microdissections and assay of aromatase activity

To assess the efficacy of fadrozole in inhibiting the activity of aromatase, we microdissected two regions of the brain that are well known to express very high levels of aromatase, the hypothalamic-preoptic area (HPOA) and the NCM, and ran on these samples an *in vitro* assay measuring aromatase activity (AA). The method used for microdissecting out the HPOA was modified for use in canaries from that used by Cornil and colleagues^64^ in quail. The brain was sectioned in 200 μm thick coronal slices with the plane of section adjusted to the stereotaxic atlas of canary^65^. Sections were mounted on frozen microscope slides and individual regions were then immediately collected by cutting them out with a scalpel. The hypothalamic/preoptic area (HPOA) was collected from the most rostral section containing the full extension of the tractus septopallio-mesencephalicus (TSM) to the most caudal section containing the end of the anterior commissure (CA). For each section, the dissection was defined by a dorsal cut performed ventral to the septum and a lateral cut at the most lateral edge of the diencephalon (defined as the junction between each telencephalon and optic lobe). In the most caudal sections an additional oblique cut was performed at the basis of the diencephalon to remove each optic lobe. A caudal mediodorsal telencephalic region containing the NCM was then collected. Both regions were delimited by a dorsoventral cut parallel to the interhemispheric line aligned to the point where the telencephalon meets the optic lobe and a ventral cut at the level of the lamina medullaris dorsalis for the rostral sections and at the level of the lamina arcopallialis dorsalis for the most caudal sections. To ensure aromatase from the hippocampus did not confound the activity present in the NCM region that was microdissected, the hippocampus was removed using a razor blade. Microdissected tissues were immediately transferred into refrigerated 1.5 ml tubes kept on dry ice and stored at −80 °C until further use.

The aromatase activity assay was performed by methods described in Cornil *et al*.^64^ with only minor modifications. The microdissected regions were homogenized with a glass homogenizer in 240 μL ice-cold buffer containing 150 mM KCL, 1 mM Na-EDTA, 10 mM Tris-HCl pH 7.2. Aromatase activity was quantified in these homogenates by measuring the tritiated water production from [1β-^3^H]-androstenedione^24^. On an ice bath, triplicate aliquots (50 μl) of homogenate were added to 50 μl of 100 nM [1β-^3^H]-androstenedione (Specific activity = 24.0 Ci/mmol) and 50 μl of buffer. To initiate the assay, 50 μl of NADPH was added so as to reach a final concentration of 1.2 mM. All these steps were conducted at 4 °C in 1.5-ml Eppendorf^®^ tubes which were then quickly capped and incubated for 20 minutes at 37 °C. The reaction was stopped by cooling the samples in an ice bath and adding 0.4 ml ice-cold 10% trichloroacetic acid containing 2% activated charcoal. After centrifugation at 1200 *g* for 15 min, supernatants were applied to small columns made of Pasteur pipettes plugged with glass beads and filled (3 cm high) with a Dowex cation exchange resin AG 50 W-X4, 100–200 mesh (Biorad, Richmond, CA). The columns were then eluted with 3 × 0.6 ml distilled water. Effluents were collected in scintillation vials and 10 ml Ecoscint A (National Diagnostics, Atlanta, GA) were finally added. Vials were counted for 3 min on a Packard Tri-Carb 1600 TR Liquid Scintillation analyzer.

For each subject an additional tube was incubated in the presence of an excess (final concentration about 40 μM) of the potent and specific aromatase inhibitor, R76713 (Racemic vorozole, Janssen Pharmaceutica, Beerse, Belgium) providing blank values of enzymatic activity. A recovery of 93 ± 2% is usually obtained from samples of 10,000 dpm tritiated water conducted throughout the entire purification procedure (incubation, centrifugation and Dowex column). Enzyme activity was expressed in total fmol h^−1^ after correction of the counts for quenching, recovery, blank values and percentage of tritium in β-position in the substrate.

### Statistical Analyses

To test the effects of fadrozole on the inhibition of aromatase in the HPOA and NCM, we conducted Bonferroni-corrected planned comparisons comparing the effects of Fadrozole at the different time points. Aromatase activity values were log-transformed. Outliers were excluded if they were beyond +/−2 standard deviations from the mean. Paired t-tests were used to assess the effects of treatment on song measures. Percentages of birds singing on each injected day were compared with the Fisher exact probability test. Effects were considered significant at p ≤ 0.05 using two-tailed statistical analyses. Effect sizes were reported as Cohen’s d for t-tests when significant differences were observed.

## Additional Information

**How to cite this article**: Alward, B. A. *et al*. Aromatase inhibition rapidly affects in a reversible manner distinct features of birdsong. *Sci. Rep*. **6**, 32344; doi: 10.1038/srep32344 (2016).

## Figures and Tables

**Table 1:** Descriptive statistics and statistical information for the effects of fadrozole injection on song features.

Statistical Information												
Day of injection, vehicle (VEH) versus fadrozole (FAD)							Baseline (BL) versus Day after FAD (DAF)					
*Measure*	*Mean* ± *SEM*	*Mean Dff* ± *SEM Diff*	*t*	*df*	*p*	*Cohen’s d*	*Mean* ± *SEM*	*Mean Dff* ± *SE Diff*	*t*	*df*	*p*	*Cohen’s d*
Latency to sing after injection (min)	VEH: 129.7 ± 47.1	114.9 ± 33.4	3.44	6	0.01*	1.30	—	—	—	—	—	—
	FAD: 244.6 ± 65.6											
% Time singing	VEH: 5.1 ± 1.1	3.2 ± 1.1	2.98	6	0.02*	1.13	BL: 11.7 ± 2.6	3.5 ± 1.7	2.05	11	0.07	—
	FAD: 1.9 ± 0.6						DAF: 8.2 ± 2.5					
Song duration (sec)	VEH: 10.0 ± 2.1	4.3 ± 1.9	2.75	6	0.03*	1.04	BL: 7.4 ± 1.2	−0.5 ± 0.8	0.7	11	0.49	—
	FAD: 5.7 ± 1.1						DAF: 7.9 ± 1.2					
Bandwidth CV	VEH: 50.0 ± 9.3	−29.8 ± 8.5	3.49	6	0.01*	1.31	BL: 53.7 ± 8.4	−2.3 ± 3.6	0.7	11	0.53	—
	FAD: 70.8 ± 14.9						DAF: 55.9 ± 7.9					

Descriptions of the different song variables are provided within the main text. Means ± Standard Error of the Mean (SEM) are shown for the two treatments. Additionally, the Mean Differences (±SEM) within-subject (*Mean Diff* ± *SEM Diff*) are shown for each comparison. Effect sizes are represented with Cohen’s d. Significant within-subject effects are written in bold with a single asterisk next to its p value. All statistical tests were two-tailed. Effects were considered significant at p ≤ 0.05. DAF = day after fadrozole.
